# Immunoproteomic Identification and Vaccine Assessment of *Trypanosoma vivax* Invariant Surface Glycoprotein

**DOI:** 10.3390/vaccines14030226

**Published:** 2026-02-28

**Authors:** Genaro Francisco Díaz, Larisa Rossini, Yael Cusinier, Diego Gustavo Arias, Iván Bontempi

**Affiliations:** 1 Laboratorio de Tecnología Inmunológica, Facultad de Bioquímica y Ciencias Biológicas, Universidad Nacional del Litoral, Santa Fe 3000, Argentinacusiniery@hotmail.com (Y.C.); 2 Laboratorio de Enzimología Molecular, Instituto de Agrobiotecnología del Litoral (CONICET-UNL), Santa Fe 3000, Argentina

**Keywords:** *Trypanosoma vivax*, invariant surface glycoproteins, vaccine candidate, immunoproteomics, trypanotolerance

## Abstract

Background: African animal trypanosomosis, caused by *Trypanosoma vivax*, remains a significant challenge to cattle health and productivity in regions where it is endemic. The development of vaccines against this parasite is particularly challenging due to its highly effective immune evasion mechanisms. Methods: An immunoproteomic approach was employed to identify *T. vivax* antigens through the immunocapture of parasite proteins using purified IgG from naturally infected sera. The objective of this strategy was to identify novel vaccine candidates, evaluated in a BALB/c murine model, aimed at promoting the induction of trypanotolerance. Results: An invariant surface glycoprotein (Uniprot code: F9WVM3, Tritryps code: TvY486_0045500), here designated TvISGAf, was selected based on its reported diagnostic relevance and its classification within the vivaxin antigen family. The protective potential of TvISGAf was evaluated in a murine model of *T. vivax* infection. Immunization with TvISGAf induced a robust antigen-specific humoral response, accompanied by a substantial cellular immune response. Following challenge, mice immunized with TvISGAf formulated with the ISPA adjuvant demonstrated enhanced control of body weight and hematocrit, and improved survival during the acute phase of infection in comparison to control group. Cytokine profiling revealed elevated levels of IFN-γ and TNF-α, accompanied by increased IL-10 production. Conclusions: Collectively, these findings demonstrate that TvISGAf formulated with ISPA confers partial protection during acute phase of infection, consistent with the induction of trypanotolerance. These results support its potential as a promising component of a multivalent vaccine strategy against *T. vivax*, and highlight the need for further evaluation prior to assessment in the bovine host.

## 1. Introduction

African animal trypanosomosis (AAT) represents a significant challenge to livestock production in sub-Saharan Africa and in regions of the Americas where *Trypanosoma vivax* has established itself independently of its original tsetse-based transmission cycle [1,2]. *Trypanosoma vivax* is the primary etiological agent of bovine trypanosomosis (BT) in South America and a significant cause of AAT in Africa [3]. The disease has been associated with substantial economic losses, including reduced productivity, veterinary expenses, and mortality. These losses have been documented in Africa [4] and Latin America [5,6]. The clinical manifestations of the disease include intermittent fever, anemia, weight loss, reduced milk production, apathy, abortions, and, in the absence of treatment, mortality rates that may reach up to 50% [7]. However, some animals develop subclinical infections [8], becoming chronic carriers that contribute to the silent transmission of the infection within the herd [9]. The available treatments, including isometamidium chloride and diminazene aceturate, present significant limitations in terms of accessibility and cost [10]. Moreover, the efficacy of these measures has been observed to decrease in the presence of drug-resistant strains that have emerged in Africa and South America [11,12,13]. In this context, the development of safe, effective, and economically viable alternatives is a high priority [14,15]. Vaccination against BT offers a promising approach to reduce reliance on trypanocidal drugs and mitigate productivity losses. However, this approach has historically been underexplored due to the complex immunobiology of trypanosomes [16,17].

A distinguishing characteristic of trypanosomes from the subgenus Salivaria is their exclusive extracellular localization within the mammalian host. This results in perpetual exposure to the host’s humoral immune system. This evolutionary pressure has led to the development of sophisticated immune-evasion mechanisms, with the expression of variant surface glycoproteins (VSGs) being the most notable example [18,19,20]. The dense VSG coat functions as a protective shield, thereby masking other membrane antigens from immune recognition. The process of immune evasion, as observed in VSG, occurs through two distinct mechanisms: the initial hydrodynamic removal of surface-bound antibodies during parasite motility [21] and antigenic variation mechanisms [22,23]. Nevertheless, the presence of several low-abundance surface proteins that undergo antigenic variation has been identified. Collectively, these are known as invariant surface glycoproteins (ISGs). These conserved proteins are regarded as promising targets for novel immunization strategies, as they may remain accessible to the immune system even in the presence of the VSG coat [24]. In recent years, ISGs have emerged as promising vaccine candidates against *T. vivax* [25,26]. The aforementioned studies evaluated a multitude of antigens and reported a decline in parasitemia during the early days post-infection, thereby reinforcing the potential of ISGs as immunogens.

For a vaccine candidate against *T. vivax*, effective control of parasitemia depends on the induction of a coordinated immune response that integrates both antibody-mediated and cell-mediated effector mechanisms. Protective immunity is contingent upon the generation of lytic antibodies as well as a robust cell-mediated response, which is typified by M1 macrophage activation and the production of pro-inflammatory cytokines such as IFN-γ and TNF-α [27,28,29,30,31,32]. These immune mechanisms are critical for parasite clearance during the acute phase of infection. However, it should be noted that prolonged exposure to high levels of pro-inflammatory cytokines can lead to immune system dysfunction, manifested by symptoms such as severe anemia and progressive body weight loss [33].

The present study sought to identify antigens recognized by the immune system of cattle naturally infected with *T. vivax* from different geographic regions and to evaluate their potential as vaccine candidates capable of inducing protective immunity. To formulate these antigens, two distinct adjuvants with divergent immunomodulatory properties were employed: ISPA [34] and MONTANIDE™ ISA 50 V2 (SEPPIC). ISPA is a biosimilar of ISCOMATRIX [35], composed of cholesterol, phospholipids, and saponin, and is known to induce a balanced humoral and cellular immune response. This adjuvant has been the subject of extensive evaluation by our research group in the context of experimental vaccines against *Trypanosoma cruzi* [36,37,38]. While *T. cruzi* infection features an intracellular stage that is critically dependent on CD8^+^ T-cell activation, *T. vivax* is an exclusively extracellular parasite. Consequently, effective immunity is predicted to depend on a coordinated interplay between humoral and cellular immune responses [16,33]. In contrast, MONTANIDE™ ISA 50 V2is a commercially available adjuvant that has been extensively utilized in cattle vaccines. It has been demonstrated to elicit balanced Th1- and Th2-type cytokine responses with notable efficacy [39,40]. The employment of these complementary adjuvants enabled the assessment of the immunogenicity and protective potential of selected *T. vivax* antigens under formulations relevant to both experimental and field-applicable vaccination strategies.

## 2. Materials and Methods

### 2.1. Sera

All positive serum samples were collected from dairy cattle, primarily of the Holstein and Jersey breeds, and were obtained through the Bovine Trypanosomiasis Diagnostic Service at the Facultad de Bioquímica y Ciencias Biológicas, Universidad Nacional del Litoral. Samples of negative serum were collected from dairy cattle that were raised in non-endemic regions of southern Argentina. These animals had no documented history of exposure to trypanosomes and exhibited no clinical signs of trypanosomosis. For the purpose of antigen identification through immunoprecipitation and proteomic analysis, sera from ten cattle naturally infected with *T. vivax* were utilized. The infection status was confirmed by PCR in accordance with the protocol described by Cortez et al. [41].

### 2.2. IgG Purification from Serum and Coupling to CNBr-Activated Sepharose

Serum samples from cattle with and without infection were subjected to salt fractionation by differential ammonium sulfate precipitation to enrich the IgG fraction. Each serum sample (10 mL) was thoroughly mixed with an equal volume of saturated ammonium sulfate solution, employing a technique of inversion to ensure homogeneity. Subsequently, the mixture was placed on ice for a period of 15 min, a process that was intended to induce the precipitation of the desired components. The mixture was then subjected to a centrifugal process at 10,000× *g* for 15 min at a temperature of 4 °C. Subsequently, the upper layer was extracted, and the resulting pellet was meticulously resuspended in approximately half of the initial volume of distilled water. Subsequently, an equivalent volume of 66% saturated ammonium sulfate solution was incorporated. The protein suspension was then subjected to an ice bath for a period of 15 min. The mixture was subsequently exposed to a centrifugal process under conditions that were identical to those previously described. The resulting serum was then removed, and the precipitate was dissolved in distilled water. This precipitation step was repeated twice. The final pellet was resuspended in 3 mL of 10 mM sodium phosphate buffer, pH 7.4. In order to eliminate any residual salts, the IgG-enriched fraction was subjected to dialysis using semipermeable membranes (Visking tubing, Cytiva, Washington, DC, USA) against a 10 mM sodium phosphate buffer, pH 7.4, at a temperature of 4 °C for a period of 3 days, with daily changes in the buffer being performed.

For the coupling process, CNBr-activated Sepharose (GE Healthcare, Chicago, IL, USA) was utilized for each IgG sample (IgG fraction from infected animals and IgG fraction from non-infected animal controls), in accordance with the manufacturer’s recommendations. In the experimental procedure, the dry CNBr-activated Sepharose was weighed out at a precise quantity of 0.5 g, and the purified IgG was measured at a range of 25–30 mg. These two elements were utilized in the coupling process, a procedure that was then assessed for its efficacy by measuring the remnant concentration of free IgG after the incubations. Subsequent to this, the conjugated beads were meticulously washed and stored in a 10 mM sodium phosphate buffer, pH 7.4, containing 0.02% (*w*/*v*) sodium azide at 4 °C.

### 2.3. Production of T. vivax Lysate

BALB/c mice were infected with *T. vivax* Y486 previously stored at −80 °C. After seven days, infected blood was collected using sodium heparin as anticoagulant, and 5 × 10^5^ *T. vivax* parasites in phosphate-buffered saline (PBS, pH 7.2) supplemented with 20 mM glucose were reinjected into three additional BALB/c mice. Blood was collected four days post-infection, and parasites were separated by centrifugation at 150× *g* for 10 min. The purified trypanosomes were resuspended in 1 mL of equilibration buffer [PBS, 1 mM EDTA, non-ionic detergent Nonidet P-40 (Sigma-Aldrich, St. Louis, MO, USA), and protease inhibitor cocktail]. Parasites were disrupted by sonication using a high-intensity ultrasonic homogenizer (PULCE 150, Benchmark, Sayreville, NJ, USA). The lysate was centrifuged at 10,000× *g* for 10 min at 4 °C, and the supernatant was collected.

### 2.4. Immunoprecipitation

For each immunoprecipitation, 1 mL of *T. vivax* lysate (protein concentration: 0.64 mg/mL, obtained as described above) was incubated with the corresponding Sepharose–IgG matrix (infection or control) under gentle agitation for 15 min at 4 °C. After washing the resins with PBS solution (three times), bound trypanosome proteins were eluted 3 times with 1 mL of 100 mM glycine-HCl (pH 2.8). The eluates were pooled and immediately neutralized with 250 µL of 200 mM Tris-HCl (pH 8.5). The eluates were then concentrated to 200 µL using a centrifugal concentrator (Sartorius, Göttingen, Germany, 20 mL capacity, 10 kDa MWCO). The concentrated fractions were stored at −20 °C.

### 2.5. Proteomic Protein Identification

Proteins eluted from the infection and control IgG immunoprecipitations were analyzed by mass spectrometry LC–MS/MS at the Mass Spectrometry Facility (Mass Spectrometry Unit of the Institute of Molecular and Cellular Biology of Rosario, UEM-IBR, Argentina) using a Thermo Scientific (Waltham, MA, USA) Q-Exactive HF mass spectrometer coupled to a nanoLC system. MS/MS spectra were processed with Proteome Discoverer 2.4 and searched against the *T. vivax* (strain Y486, UniProt), *Bos taurus*, and common contaminant databases. Searches allowed up to two missed trypsin cleavages and included carbamidomethylation of cysteine as a static modification, and methionine oxidation and N-terminal acetylation—including Met-loss variants—as variable modifications. Protein identifications and peptide intensity data were obtained using standard Orbitrap search parameters.

### 2.6. Protein ID Mapping and Gene Ontology Annotation

Protein identifiers were mapped and functionally annotated using the UniProt database. The initial list of protein IDs was submitted to the UniProt ID mapping tool to retrieve standardized UniProt accession numbers and associated annotations. Gene Ontology (GO) annotations were retrieved directly from UniProt database and classified into the three main GO domains: Biological Process (BP), Molecular Function (MF), and Cellular Component (CC). For proteins associated with multiple GO terms within a given domain, all annotations were retained and treated independently in downstream analyses. Proteins lacking GO annotations in UniProt database were classified as “Uncharacterized protein” for the corresponding GO domain. The distribution of GO slim categories for each ontology (BP, MF, and CC) was visualized using pie charts, representing the relative contribution of each functional category.

### 2.7. Expression of Recombinant TvISGAf

The immunization protocols involved the production of the TvISGAf recombinant protein as a 6His–MBP fusion. This process was carried out in accordance with the expression and purification procedures previously described by our research group [42]. To evaluate both humoral and cellular immune responses, TvISGAf was expressed as a fusion with red fluorescent protein (RFP). The recombinant production and purification of the 6His–RFP–TvISGAf protein were carried out as follows. The pGEM-T Easy/TvISGAf construct was then digested with BglII and HindIII, and the purified insert was ligated into the pRED plasmid—a custom vector derived from pET32a designed to generate 6His–RFP fusion proteins—which had been digested with BamHI and HindIII (Promega, Fitchburg, WI, USA). Competent *E. coli* BL21 (DE3) cells were transformed with the resulting construct and selected on lysogeny broth agar plates supplemented with 100 μg/mL ampicillin. The expression of the recombinant protein was induced with 0.25 mM IPTG for a period of 16 h at a temperature of 23 °C. Protein purification was performed by immobilized metal affinity chromatography (IMAC) with a 1 mL Ni^2+^ HiTrapTM chelating HP column (GE Healthcare), following our previously reported protocol [41]. Briefly, the bacterial pellet was suspended in a binding buffer composed of 20 mM Tris–HCl (pH 7.5), 400 mM NaCl, and 10 mM imidazole. The bacterial pellet was subsequently lysed by sonication using a Vibra-cell™ VCX-600 ultrasonic processor (Sonics & Materials Inc., Newtown, CT, USA). Following cell disruption, the suspension was subjected to centrifugation at 10,000× *g* for a duration of 30 min, resulting in the clarification of the suspension. Subsequently, the protein was applied to an affinity column that had been pre-equilibrated with the appropriate buffer. After undergoing extensive washing with ten column volumes of binding buffer to remove non-specifically bound material, the target protein was recovered using an elution buffer containing 20 mM Tris–HCl (pH 7.5), 400 mM NaCl, and 300 mM imidazole. The eluted fractions with the highest purity were then combined, concentrated, and dialyzed against 20 mM Tris–HCl (pH 8.0), 200 mM NaCl, and 1 mM EDTA. The final preparation was supplemented with 20% (*v*/*v*) glycerol and stored at −80 °C.

### 2.8. Immunization Using Recombinant TvISGAf Protein in Experimental Mice

BALB/cCmedc female mice (6 to 8 weeks of age) were utilized in all experimental procedures. These mice were obtained from the Centro de Medicina Comparada, ICIVET-CONICET, UNL, Esperanza, Santa Fe, Argentina. The experimental protocols were developed in accordance with the ARRIVE guidelines and were carried out in accordance with the National Research Council’s Guide for the Care and Use of Laboratory Animals. The aforementioned protocols were formally endorsed by the Facultad de Bioquímica y Ciencias Biológicas’s (UNL, Argentina) Advisory Committee of Ethics and Research Safety (CAESI) through Resolution n◦ CE2021-02. Five groups of mice (*n* = 5 per group) were utilized to assess the immunogenicity of TvISGAf formulated with two distinct adjuvants: ISPA [34] and MONTANIDE™ ISA 50 V2 (Seppic, La Garenne-Colombes, France). Mice received three subcutaneous immunizations administered two weeks apart. Each dose contained one of the following formulations: (a) 10 µg of TvISGAf combined with 2 µL of ISPA (TvISGAf–ISPA), (b) 10 µg of TvISGAf in 50 µL PBS with 50 µL de MONTANIDE™ ISA 50 V2 (TvISGAf-Mont), (c) 2 µL of ISPA (ISPA), (d) 50 µL PBS with 50 µL de MONTANIDE™ ISA 50 V2 (Mont), (e) vehicle control (PBS). Blood samples were collected on days 7, 21, and 35 after immunization to assess antigen-specific anti-TvISGAf antibody responses by ELISA, expressed as optical density (OD) values.

### 2.9. Assessment of Humoral Immune Response

Microtiter plates (Greiner Bio-One, Kremsmünster, Austria) were coated with 0.5 µg/well of TvISGAf in 50 mM carbonate–bicarbonate buffer (pH 9.6) and subsequently blocked with PBS containing 5% (*w*/*v*) milk. Serum samples collected after immunization were diluted 1:100 in PBS with 1% (*w*/*v*) milk and added in duplicate to the TvISGAf protein-coated wells. TvISGAf-specific antibodies were detected using goat anti-mouse IgG (1:10,000; Sigma-Aldrich, USA) or anti-mouse IgG1 and IgG2a (1:10,000; Abcam, Cambridge, UK). After incubation with 50 µL of ready-to-use TMB substrate (Invitrogen), the enzymatic reaction was stopped with 50 µL of 1 M sulfuric acid, and absorbance was recorded at 450 nm using an ELISA plate reader (Bio-Tek Instruments, Winooski, VT, USA). The cut-off value was established as the mean optical density (OD) of pre-immune sera plus two standard deviations.

### 2.10. Delayed-Type Hypersensitivity (DTH) Test

The DTH response was evaluated by intradermal injection of 5 µg of TvISGAf into the left hind footpad 10 days after the final immunization. The thickness of the footpad was measured immediately prior to antigen administration and again 48 h post-challenge using a Vernier caliper (Stronger, Linyi, Shandong, China). The response was expressed as the increase in footpad thickness (mm) attributable to antigen stimulation.

### 2.11. Spleen Cell Culture and Cytokine Determination

Seven days after the completion of the immunization protocol, the mice were euthanized so that the ex vivo cytokine production by the splenocytes could be evaluated. The splenocytes were stimulated with recombinant TvISGAf. Spleens were aseptically collected and homogenized, and red blood cells were removed using lysis buffer (Sigma). The resulting splenocytes were resuspended in RPMI 1640 (Gibco, Grand Island, New York, NY, USA) supplemented with 10% (*v*/*v*) FBS, penicillin (100 µg/mL), streptomycin (100 U/mL), and 0.4 mM 2-mercaptoethanol. Cells (2 × 10^6^/mL per well) were seeded in 48-well plates (Nunc, Roskilde, Denmark) and cultured in complete RPMI alone or stimulated with TvISGAf (10 µg/mL) or Concanavalin A (2.5 µg/mL), the latter serving as a positive control. Following a 48 h incubation period, the culture supernatants were collected, and the levels of IFN-γ, IL-10, and TNF-α were quantified by ELISA. The quantification process followed the manufacturer’s instructions (Mouse IFN-γ/IL-10/TNF-α Uncoated ELISA Kit, Invitrogen, Carlsbad, CA, USA). The detection limits for IFN-γ, IL-10, and TNF-α were determined to be 16 pg/mL, 32 pg/mL, and 8 pg/mL, respectively.

### 2.12. Infection Protocol and Protection Parameters

The same immunization protocol was evaluated using ISPA and MONTANIDE™ ISA 50 V2 as adjuvants. Subsequent to the completion of the final immunization, the animals were infected intraperitoneally with 1000 bloodstream forms of the *T. vivax* Y486 strain, which was provided by Dr. Greif Gonzalo of the Institut Pasteur de Montevideo. The time to parasite establishment in immunized mice and their survival rates were used to assess the degree of post-challenge protection. In this murine infection model, non-immunized mice infected with *T. vivax* typically exhibit approximately 50% mortality between days 8 and 12 post-infection. Consequently, the protection parameters were monitored for a period of 14 days following the infection. Parasitemia, hematocrit levels, and body weight were recorded at two-day intervals. As delineated by Guegan et al. [43], the hematocrit level was measured in accordance with established protocols. In summary, blood samples were obtained via tail bleed and placed in 80 µL Na-heparin–coated capillary tubes. Subsequent to this, the tubes were sealed, subjected to centrifugation (2000× *g*, 10 min), and analyzed to determine packed cell volume (PCV). Alternatively, 5 µL of tail blood was lysed in 100 µL of distilled water, and the OD405 of a 40-fold dilution was determined. Parasitemia was quantified by examining 25 microscopic fields at a final magnification of 400×. The estimation of parasite concentration was then conducted by applying a conversion factor derived from the microscope field number, the dimensions of the coverslip, and the sample volume.

### 2.13. Statistical Analysis

The subsequent analysis of the data employed nonparametric statistical methods. The assessment of global group differences was conducted using the Kruskal–Wallis test, followed by Mann–Whitney U tests for predefined pairwise comparisons. To account for the multiple testing inherent in such analyses, exact *p*-values for these pairwise comparisons were adjusted using the Holm method. Survival analyses were performed using the Mantel–Cox log-rank test. Statistical analyses were conducted using GraphPad Instat 4.0 (GraphPad, San Diego, CA, USA). The significance thresholds were established at *p* < 0.05 (*) and *p* < 0.01 (**).

## 3. Results

### 3.1. Antigen Identification

To identify candidate antigens, pooled serum samples were prepared from both naturally infected cattle and healthy, uninfected controls. Each serum pool comprised ten samples from *T. vivax*-infected animals and ten from non-infected animals. The IgG fractions were subjected to ammonium sulfate precipitation, followed by immobilization on CNBr–activated Sepharose beads. Subsequently, equivalent amounts of infection-IgG–Sepharose and control-IgG–Sepharose were incubated with equal quantities of a *T. vivax* whole-cell lysate. The proteins specifically retained on the IgG columns were subsequently eluted under low-pH conditions. Immunocaptured proteins were then subjected to mass spectrometry for identification.

**Figure 1:**
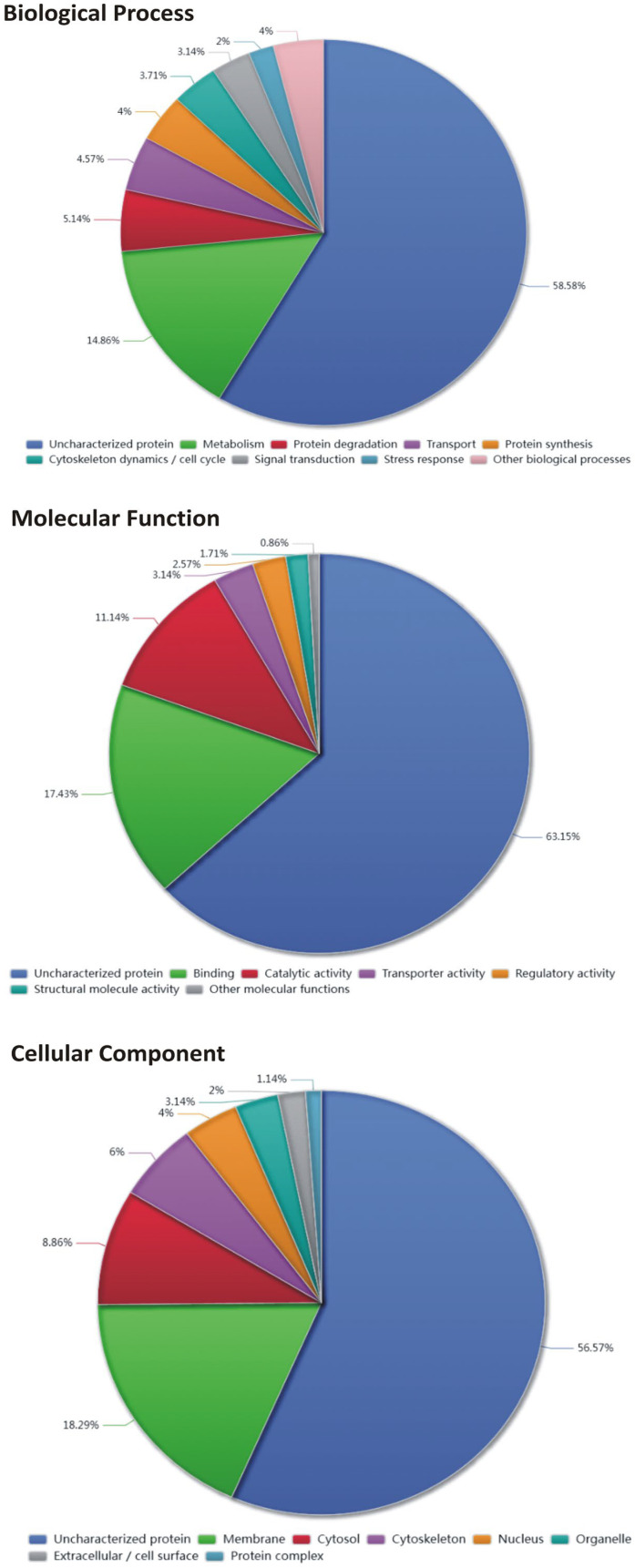
Gene ontology analysis of the identified proteins. Functional distribution of the analyzed proteins according to Gene Ontology (GO) annotations, represented by pie charts for the three main ontologies: Biological Process, Molecular Function, and Cellular Component. Individual GO terms were initially counted and subsequently grouped into high-level functional categories (GO slim) to reduce semantic redundancy and facilitate biological interpretation. This reduction was performed in an ontologically specific manner, using process categories for Biological Process, molecular activity categories for Molecular Function, and subcellular localization categories for Cellular Component. Proteins without a valid GO annotation or without a functional assignment were grouped into the “Uncharacterized protein” category. The percentages indicate the relative proportion of each category with respect to the total number of annotations considered in each ontology.

Gene Ontology (GO) analysis of the proteins exclusively identified in the positive sera group (Figure 1) revealed a functional landscape characterized by a high proportion of proteins lacking functional annotation, reflecting the limited characterization of the *T. vivax* proteome. Among the annotated proteins, functional categories were predominantly enriched in metabolic processes, followed by protein degradation, transport, and cytoskeletal-associated processes. This finding suggests a pronounced bias toward core cellular functions. The ontology analysis revealed a predominant association with membrane-related compartments, followed by cytosolic and cytoskeletal localizations. This finding underscores the importance of membrane-associated and structural proteins in the analyzed dataset. Of particular interest is the identification of invariant surface glycoproteins (Table 1). Surface-exposed glycoproteins that exhibit limited sequence variability are of particular interest as vaccine antigens. Indeed, proteins localized to cellular membranes or extracellular interfaces are inherently accessible to the host immune system and therefore represent attractive candidates for humoral immunity–based vaccine strategies. The prevalence of membrane and surface-associated categories suggests the presence of proteins with exposed epitopes capable of eliciting antibody-mediated responses, including neutralization or opsonization. Consequently, these findings support the prioritization of membrane-associated proteins as potential antigen candidates for further experimental validation in the context of vaccine development.

### 3.2. Expression and Purification of Recombinant TvISGAf Protein

**Figure 2:**
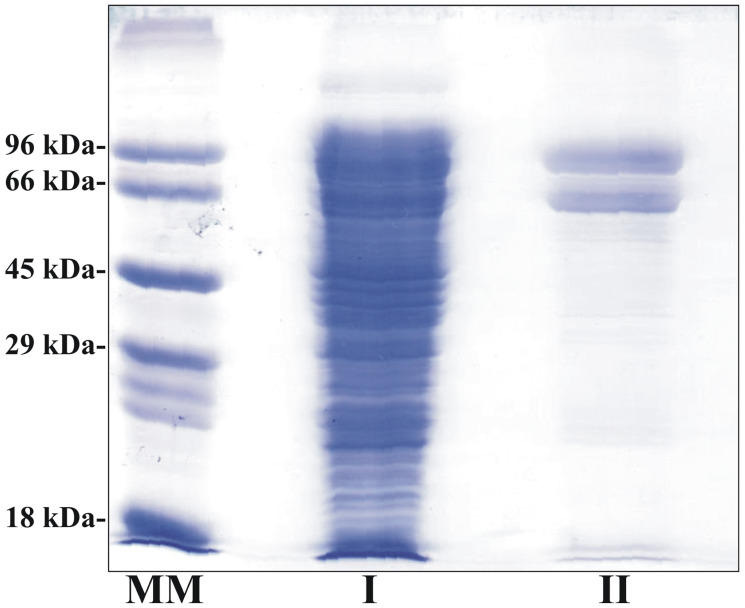
Expression and purification of the recombinant 6HisRFP–TvISGAf protein. Proteins were separated on a 12% (*w*/*v*) SDS–PAGE gel and visualized by Coomassie staining. MM: molecular weight marker; I: total soluble fraction from IPTG-induced *E. coli* BL21 (DE3) carrying the 6HisRFP–TvISGAf protein; II: purified 6HisRFP–TvISGAf recombinant protein.

In order to conduct vaccine studies, a recombinant antigen as His-tag fusion protein was developed using the plasmids pET28c and pET22b. Nonetheless, subsequent to the evaluation of the various induction conditions (*E. coli* strains, culture media, induction time, and temperature), the expression of the recombinant protein remained undetected. Consequently, drawing from our extant research [42], we undertook the synthesis of this antigen as an N-terminal fusion protein to 6HisMBP. For ELISA, DTH, and splenocyte culture assays, TvISGAf was expressed as a 6His–RFP fusion protein. The amino acid sequence is presented in Figure S1. The N-terminal 6His-RFP fusion protein enhanced the production and solubility of the recombinant antigen in *E. coli* cells (Figure 2). SDS-PAGE analysis confirmed that the recombinant protein was obtained with a high degree of electrophoretic purity (Figure 2). However, an additional band of lower apparent molecular weight and comparable intensity was detected). This secondary band is consistent with the co-purification of an N-terminally tagged truncated polypeptide, which may arise during expression. The final protein preparations were stored at −80 °C and demonstrated stability for a period of at least eight months.

### 3.3. Humoral Response Following Immunization

**Figure 3:**
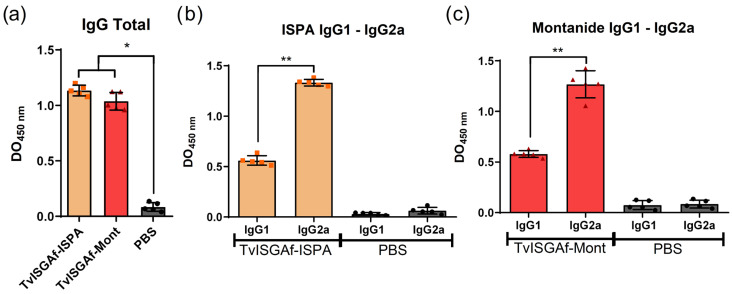
Humoral immune response: BALB/c mice were immunized with TvISGAf–ISPA, TvISGAf–Mont, or PBS. Seven days after the final immunization, serum samples were analyzed by ELISA to quantify TvISGAf-specific antibodies. (**a**) Total IgG levels. (**b**) IgG1 and IgG2a subclasses in mice immunized with TvISGAf–ISPA. (**c**) IgG1 and IgG2a subclasses in mice immunized with TvISGAf–Mont. Results are shown as mean ± SD from three independent experiments (*n* = 5/group). * *p* < 0.05; ** *p* < 0.01.

The administration of recombinant TvISGAf proteins resulted in the induction of a robust and antigen-specific humoral immune response in immunized mice, as illustrated in Figure 3. TvISGAf-immunized animals demonstrated significantly higher levels of antigen-specific total IgG compared with PBS control groups (*p* = 0.0158, PBS vs. TvISGAf-ISPA; *p* = 0.0158, PBS vs. TvISGAf-Mont; Figure 3a). A comprehensive analysis of IgG subclasses was conducted, revealing a predominance of IgG2a over IgG1 (*p* = 0.0079, IgG1 vs. IgG2a TvISGAf-ISPA; *p* = 0.0079, IgG1 vs. IgG2a TvISGAf-Mont; Figure 3b,c). This finding is consistent with the induction of complement-activating and potentially neutralizing antibodies, suggesting a Th1-biased humoral response. Notably, this profile was observed with both adjuvant formulations.

### 3.4. DTH and Serum Cytokine Responses Following Immunization

**Figure 4:**
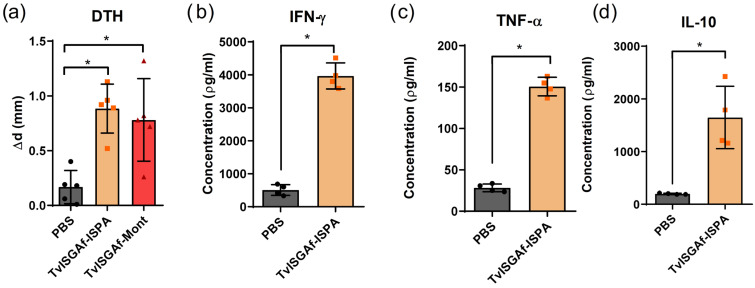
Cellular immune response: (**a**) Delayed-type hypersensitivity (DTH) response assessed 12 days after the final immunization. Footpad thickness was measured immediately before and 48 h after intradermal injection of 5 µg TvISGAf. Results are expressed as Δ (post- minus pre-injection values). (**b**–**d**) Cytokine production by splenocytes stimulated ex vivo with TvISGAf for 48 h. Concentrations of (**b**) IFN-γ, (**c**) TNF-α, and (**d**) IL-10 in culture supernatants were quantified by ELISA. Data represent mean ± SD (*n* = 5/group). * *p* < 0.05.

A delayed-type hypersensitivity (DTH) footpad test was conducted to assess the in vivo cell-mediated immune response elicited by the TvISGAf–ISPA and TvISGAf-Mont formulations. As demonstrated in Figure 4a, a substantial augmentation in footpad thickness was evident in TvISGAf-immunized mice on day 12 following the final immunization, in comparison to the PBS group (*p* = 0.0158, TvISGAf–ISPA; *p* = 0.0159, TvISGAf-Mont), signifying a robust cellular DTH response.

**Figure 5:**
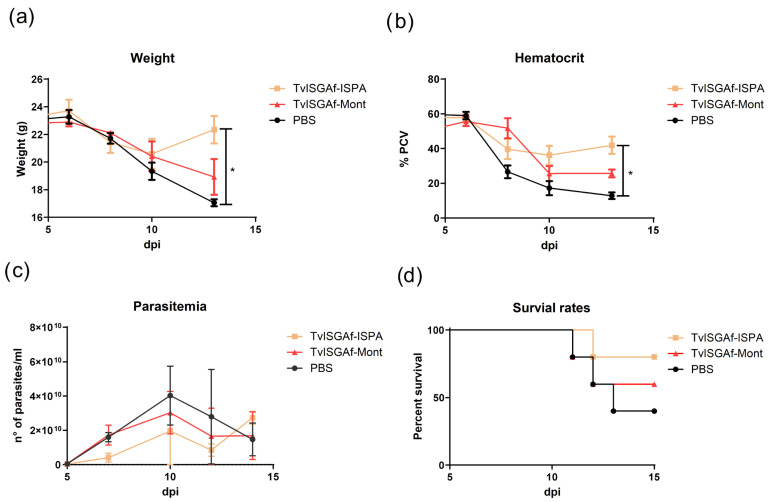
Effects of vaccination on parasitemia, anemia, body weight, and survival following experimental *T. vivax* infection. BALB/c mice immunized with TvISGAf–ISPA, TvISGAf–Mont, or PBS (n = 5/group) were challenged intraperitoneally with 1000 bloodstream forms of the *T. vivax* Y486 strain 14 days after the final immunization. (**a**) body weight, (**b**) hematocrit expressed as packed cell volume (PCV), (**c**) parasitemia, and (**d**) survival rates. Data shown correspond to one representative experiment of three performed independently. * *p* < 0.05.

To further characterize the cellular immune profile, splenocytes collected 7 days after the final immunization from the TvISGAf–ISPA and PBS groups were stimulated ex vivo with TvISGAf, and cytokine production (IFN-γ, TNF-α, and IL-10) was quantified (Figure 4b–d). Splenocytes derived from mice immunized with TvISGAf–ISPA exhibited a marked increase in the production of IFN-γ, TNF-α, and IL-10 when compared to those derived from mice immunized with PBS (*p* = 0.0286, IFN-γ; *p* = 0.0286, TNF-α; *p* = 0.0294, IL-10; Figure 5b–d). This finding indicates the activation of both pro-inflammatory and regulatory immune responses.

### 3.5. Post-Challenge Outcomes

Subsequent to the infection, the protection parameters were monitored for a period of 14 days (Figure 5). To ensure clarity of visualization, the control groups that received immunizations with MONTANIDE™ ISA 50 V2 or ISPA adjuvant alone were not included in the primary figure. This decision was made because their infection kinetics did not differ from the PBS control group. The complete dataset for these control groups is provided in the Supplementary Materials (Figure S2). During the initial phase of infection, all groups demonstrated a progressive decline in body weight and hematocrit (Figure 5a,b). However, from day 10 to day 14 post-infection, mice immunized with the TvISGAf–ISPA formulation exhibited a significant increase in body weight (*p* = 0.0214) and hematocrit values (*p* = 0.016), suggesting partial recovery at day 13 of these physiological parameters. The initial peak of parasitemia was identified on day 10 post-infection across all experimental groups. It is noteworthy that the TvISGAf–ISPA group exhibited a swift decline in parasitemia within two days following the peak, while animals in the TvISGAf–Mont and PBS control groups required approximately four days to regulate parasitemia levels. During this interval, the mortality rate in the PBS group reached 60%, while the TvISGAf–ISPA–immunized group exhibited a markedly lower mortality rate of 20% (Figure 5d). Notwithstanding these initial protective effects, no substantial disparities in long-term survival were observed among the experimental groups during the extended follow-up period.

## 4. Discussion

In order to identify novel vaccine candidates, the present study focused on antigens that are naturally targeted by the immune response of cattle infected with *T. vivax*. It was hypothesized that such antigens are likely to be exposed and immunologically relevant during natural infection. To this end, an immunoproteomic approach was employed, which has previously been used to identify trypanosome antigens using sera from African hosts infected with *Trypanosoma* spp. [44,45,46]. In the present study, this strategy was employed to capture parasite antigens using antibodies obtained from cattle naturally infected with *T. vivax* and sampled from different regions of Argentina. This methodology enabled the identification of more than 350 antigens. Gene ontology analysis revealed that a substantial fraction of the analyzed proteins lacked functional annotation, underscoring the current limitations in the characterization of the *T. vivax* proteome and highlighting the presence of potentially novel or poorly studied proteins [47]. Among the annotated fraction, the identified proteins were predominantly associated with core cellular functions, including metabolic processes, catalytic activities, and protein–protein interactions. This finding reflects a functional profile consistent with essential biological processes. A notable finding was the preponderance of membrane-associated proteins, including invariant surface glycoproteins, within the Cellular Component ontology. These proteins are of particular significance within the context of vaccine development. Surface-exposed glycoproteins that exhibit limited variability has been identified as promising targets for humoral immune responses. The identification of such molecules supports their prioritization as candidates for further immunological and functional evaluation.

Among the identified antigens, the invariant surface glycoprotein (Tritryps code: TvY486_0045500 or Uniprot code: F9WVM3) was selected, which was previously described by Fleming et al. [44]. Although in *T. brucei* ISGs are largely masked by the dense VSG coat, *T. vivax* exhibits notable structural differences, including reduced levels of VSG expression and a lower diversity of VSG genes [48]. This less compact surface organization may favor increased exposure of ISGs, facilitating their recognition by the host immune system during the infection and positioning them as attractive targets for vaccine strategies [17]. Fleming et al. [44] evaluated the diagnostic potential of the TvISGAf protein and demonstrated its strong recognition by sera from cattle naturally infected in Africa. Subsequently, our group also assessed the diagnostic performance of this protein using bovine sera from Argentina, obtaining satisfactory diagnostic performance [42]. The presence of antibodies to the TvISGAf antigen in the sera of American cattle serves to underscore the antigen’s representativeness and biological relevance in the life cycle of *T. vivax*. It is noteworthy that this antigen maintains its conservation and immunological recognizability despite the substantial genome degradation observed in American *T. vivax* strains [49,50].

More recently, Romero-Ramírez et al. [26] described a new family of invariant antigens in *T. vivax* through an extensive analysis of surface glycoproteins. This family was designated as vivaxin, and TvISGAf was identified as a constituent of this family. In that study, the classification of vivaxin family members as vaccine candidates was based on multiple integrated criteria, including structural, evolutionary, and expression-related metrics such as gene length, phylogenetic distribution, and transcriptomic expression profiles. These analyses indicated that TvISGAf is as a promising vaccine candidate due to its high scores across the evaluated parameters [26]. However, it is important to acknowledge that this study did not involve experimental in vivo evaluation of this antigen in an infection model. In this context, the analysis discussed above provides further support for this candidate, as it represents a point of convergence between independent immunologically driven empirical approaches, such as antigen capture and proteomics, and bioanalytical and bioinformatic strategies. Consequently, the protective capacity of TvISGAf in this study, was evaluated using a murine model of *T. vivax* infection. The formulation of TvISGAf involved two adjuvants, each exhibiting a distinct immunomodulatory profile. ISPA and MONTANIDE™ ISA 50 V2. The evaluation of the humoral response induced by TvISGAf revealed a marked predominance of IgG2a antibodies, with levels more than twice those of IgG1 in formulations with both ISPA and MONTANIDE™ ISA 50 V2 (Figure 3). This IgG2a bias is of particular relevance due to its association with enhanced macrophage activation via Fc receptors, thereby promoting effector mechanisms such as phagocytosis and antibody-dependent cell-mediated toxicity [51]. Furthermore, delayed-type hypersensitivity (DTH) assessment following antigen re-exposure demonstrated that both adjuvanted formulations induced significantly stronger responses in comparison to the control group (Figure 4). This finding indicates effective activation of cellular immunity, evidenced by increased late-phase inflammation attributable to lymphocyte recruitment and trafficking at the inoculation site.

In developing a vaccine against trypanosome infection, our objective was to pursue an antidisease strategy, aimed at neutralizing key pathogenic factors of the parasite rather than achieving complete parasite elimination [52]. Consequently, the present study centered on the induction of trypanotolerance in vaccinated individuals. Trypanotolerance is defined as the capacity of cattle to survive infection while maintaining acceptable levels of productivity despite the presence of trypanosomes [53]. The evaluation of this phenotype can be facilitated by the integration of various parameters, including parasitemia, anemia, and body weight variation. Collectively, these parameters offer a comprehensive reflection of the severity of the disease and the resilience of the host [54]. Consequently, TvISGAf, formulated in conjunction with ISPA, was identified as a vaccine candidate exhibiting considerable promise. This candidate effectively mitigated body weight loss and hematocrit decline during the initial 14 days of infection (Figure 5). Furthermore, survival analysis revealed that during the evaluation period, up to 80% of mice in the TvISGAf–ISPA group remained alive, whereas survival in the control groups declined to approximately 40% (Figure 5d). This approach aligns with those previously employed in related studies [25,26], which conducted analyses over relatively brief post-infection periods. Although there is evidence that MONTANIDE™ ISA 50 V2adjuvant can induce a balanced immune response [40], it has also been shown to promote a durable humoral response accompanied by strong IFN-γ activation [39]. This bias toward a markedly proinflammatory response may have contributed to immune dysregulation, rendering the immunized animals as susceptible to infection as the control groups (Figure 5). In contrast, the ISPA-formulated vaccine demonstrated consistent enhancement across the evaluated parameters. This adjuvant contains a saponin with a structure similar to that of the saponin used in Quil A. In the evaluation of vaccine candidates conducted by Autheman et al. [25], the strongest protective effects of the IFX antigen were achieved when it was formulated with Quil A. Moreover, a recent study comparing both adjuvants in an experimental vaccine against *T. cruzi* demonstrated that, although both formulations induced a balanced Th1/Th2 immune profile, the ISPA adjuvant promoted higher IL-10 production than Quil A [55]. In this context, it is imperative to acknowledge that an effective and safe immune response must encompass immunoregulatory mechanisms capable of constraining excessive inflammation [56]. Immunoregulatory cytokines have been demonstrated to exert a pivotal function in the modulation of disease progression. Research conducted on trypanotolerant animals has demonstrated that the induction of IL-10 facilitates the polarization of macrophages toward an M2 phenotype, thereby reducing tissue damage and immunopathology without compromising parasite control [57,58,59]. This cytokine balance is critical for the establishment of trypanotolerance [33]. In accordance with these findings, an examination of cytokine production in splenocyte cultures from TvISGAf–ISPA–immunized mice revealed substantial production of IFN-γ and TNF-α, accompanied by a notable increase in IL-10 compared with the PBS group (Figure 4). When considered as a whole, these results are consistent with an immunological profile compatible with the development of trypanotolerance, as described in cattle naturally resistant to infection [60,61]. However, it is imperative to acknowledge that these findings were obtained in a murine model, which differs considerably from the bovine host with regard to immunobiology and infection kinetics. Consequently, while the observed phenotype resembles trypanotolerance, its interpretation must be made with caution, and validation in the natural host remains essential to confirm the biological relevance of these responses.

In summary, the efficacy of this intervention is supported by several observations, the preservation of body weight and hematocrit levels, and improved survival during the acute phase of infection. Collectively, these findings position the TvISGAf–ISPA formulation as a promising vaccine candidate capable of inducing trypanotolerance rather than sterile immunity. Given the multifactorial nature of trypanosomosis pathogenesis, it is likely that TvISGAf will need to be combined with additional antigens targeting complementary parasite mechanisms to further enhance protection. Consequently, the evaluation of multicomponent vaccine strategies in the natural bovine host constitutes a critical subsequent step.

## 5. Conclusions

An immunoproteomic screening strategy, in conjunction with functional vaccination assays, was utilized for the identification and characterization of TvISGAf, an invariant surface glycoprotein of *T. vivax*, as a novel vaccine antigen. TvISGAf is naturally recognized by antibodies from infected cattle and belongs to the recently described vivaxin family, underscoring its biological relevance. The immunization of BALB/c mice, particularly with the TvISGAf–ISPA formulation, induced a robust humoral and cellular immune response, evidenced by increased IgG2a levels and the production of IFN-γ, TNF-α, and IL-10. In the aftermath of an experimental challenge, the vaccinated mice exhibited indications of partial protection during the acute phase of infection. This observation was substantiated by an improvement in hematocrit values, the preservation of body weight, and an enhancement in survival rates. These results signify a substantial preliminary stage in the process of translating the findings into the natural bovine host. In the final analysis, validation in cattle will be essential to determining the field applicability and true protective potential of this antigen.

## Figures and Tables

**Table 1:** Examples of *T. vivax* cell membrane-associated proteins identified by immunocapture. The protein codes presented are from Uniprot (https://www.uniprot.org/ 15 November 2025). TvISGAf (TriTryps code: TvY486_0045500, Uniprot code: F9WVM3) is highlighted in bold.

Uniprot Code	Protein Names	Length (AA)
F9WKR3	65 kDa invariant surface glycoprotein	629
F9WN04	Invariant surface glycoprotein	347
F9WUQ7	adenylate cyclase (EC 4.6.1.1) (ATP pyrophosphate-lyase) (Adenylyl cyclase)	1236
**F9WVM3**	**65 kDa invariant surface glycoprotein**	**400**
G0TT75	Nodulin-like domain-containing protein	373
G0TT88	Transmembrane 9 superfamily member	645
G0TTX6	Putative serine-palmitoyl-CoA transferase (EC 2.3.1.50)	101
G0TUX6	Putative metal-ion transporter	435
G0TVZ6	Endonuclease/exonuclease/phosphatase domain-containing protein	614
G0U000	EF-hand domain-containing protein	576
G0U352	Enriched in surface-labeled proteome protein 9	558
G0U4E1	Putative phosphatidic acid phosphatase	330
G0U4F4	Putative aquaporin 3	293
G0U4G0	Putative phosphatidic acid phosphatase protein	327
G0U4K9	J domain-containing protein	384
G0U990	Putative GPI transamidase component, Tta1	352
G0UAU8	C3H1-type domain-containing protein	830
G0UC72	carbonic anhydrase (EC 4.2.1.1)	423
G0UD80	Putative intergrin alpha chain protein	620

## Data Availability

All data used in the analyses are provided within this article and its Supplementary Materials. For any additional questions, please reach out to the authors responsible for correspondence.

## Supplementary Materials

The following supporting information can be downloaded at: https://www.mdpi.com/article/10.3390/vaccines14030226/s1. Table S1: The list of Ab (−) immunoprecipitated proteins analyzed by MS is presented in “proteins-negative.xlsx” file. Table S2: The list of Ab (+) immunoprecipitated proteins analyzed by MS is showed in “proteins-positive.xlsx” file. Table S3: The gene ontology analysis of exclusive Ab (+) immunoprecipitated proteins is presented in “GO-proteins-positive exclusive.xlsx” file. Table S4: The list of exclusive Ab (+) immunoprecipitated membrane-proteins analyzed by MS is showed in “positive exclusive-membrane proteins.xlsx” file. Figure S1: Full amino acid sequence of recombinant 6HisRFP-TvISGAf. The sequence of the fusion-tag is highlighted in green, and the *T. vivax* proteins are indicated in yellow. Figure S2: Effects of vaccination on parasitemia, anemia, body weight, and survival following experimental *T. vivax* infection in controls groups. BALB/c mice immunized with ISPA, Montanide, or PBS (*n* = 5/group) were challenged intraperitoneally with 1000 bloodstream forms of the *T. vivax* Y486 strain 14 days after the final immunization. (a) body weight, (b) hematocrit expressed as packed cell volume (PCV), (c) parasitemia, and (d) survival rates. Data shown correspond to one representative experiment of three performed independently.
