# Supplementary material for: Immunoproteomic Identification and Vaccine Assessment of Trypanosoma vivax Invariant Surface Glycoprotein
Source: Vaccines (Basel). 2026 Feb 28;14(3):226. doi: 10.3390/vaccines14030226 (PMC13030271; doi:10.3390/vaccines14030226)
Supplement: Supplementary file 1 [file vaccines-14-00226-s001.zip › vaccines-4125853-supplementary figures.pdf]

SUPPLEMENTARY DATA

>TvISGAf (6HisRFP-fusion protein of 646 amino acid and 72.1 kDa)

MHHHHHHASSEDVIKEFMRFKVRMEGSVNGHEFEIEGEGEGRPYEGTQTAKLKVTKGGPLPFAWD
ILSPQFQYGSKAYVKHPADIPDYSKLSFPEGFKWERVMNFEDGGVVTVTQDSSLQDGEFIYKVKLRG
TNFPSDGPVMQKKTMGWEASTERMYPEDGALKGRDQDEAEAEGRRPLRRRGQDHLHGQEARAA
AGAYKTDIKLDITSHNEDYTIVEQYERAEGRHSTGARSDYDIPTTGTENLYFQSASGSDHVLLNMQM
RRYGPPLFVAALCLIDVVLCQASSYENEIARALCKMGSTHRRMSMVFGVLQQRISKTDDTINGLETD
LWKLKKAGLPDEKYQEVNDKVINVTGSVSLVTNAVKVAQKKLEEFIEKVKTEHYNDHYLKLEDRK
FGESVSNCRDWATYNEETPDKLRKKLESGLKTLEAWATEESNEWEKEQKEVESDLLSKENRNSLQ
YGTLHTAFKDLVKSMMVELTTVSFYMPKALEGVPGADAAVNEARKFVVVAMANECQSVASEAAAS
EEKQAQCEKLNKKLQEIKEKKRQAIGGDSEGPKSSDAKSTDATPTSSASQKVIVEEVLDSADGDELM
ELVQTADKPSAANNSKLSPTNLALAISIPVALVLIGAAVFLVMRRRTAEKVVPTI

Figure S1: Full amino acid sequence of recombinant 6HisRFP-TvISGAf. The sequence of the fusion-tag is highlighted in green, and the *T. vivax* proteins are indicated in yellow.

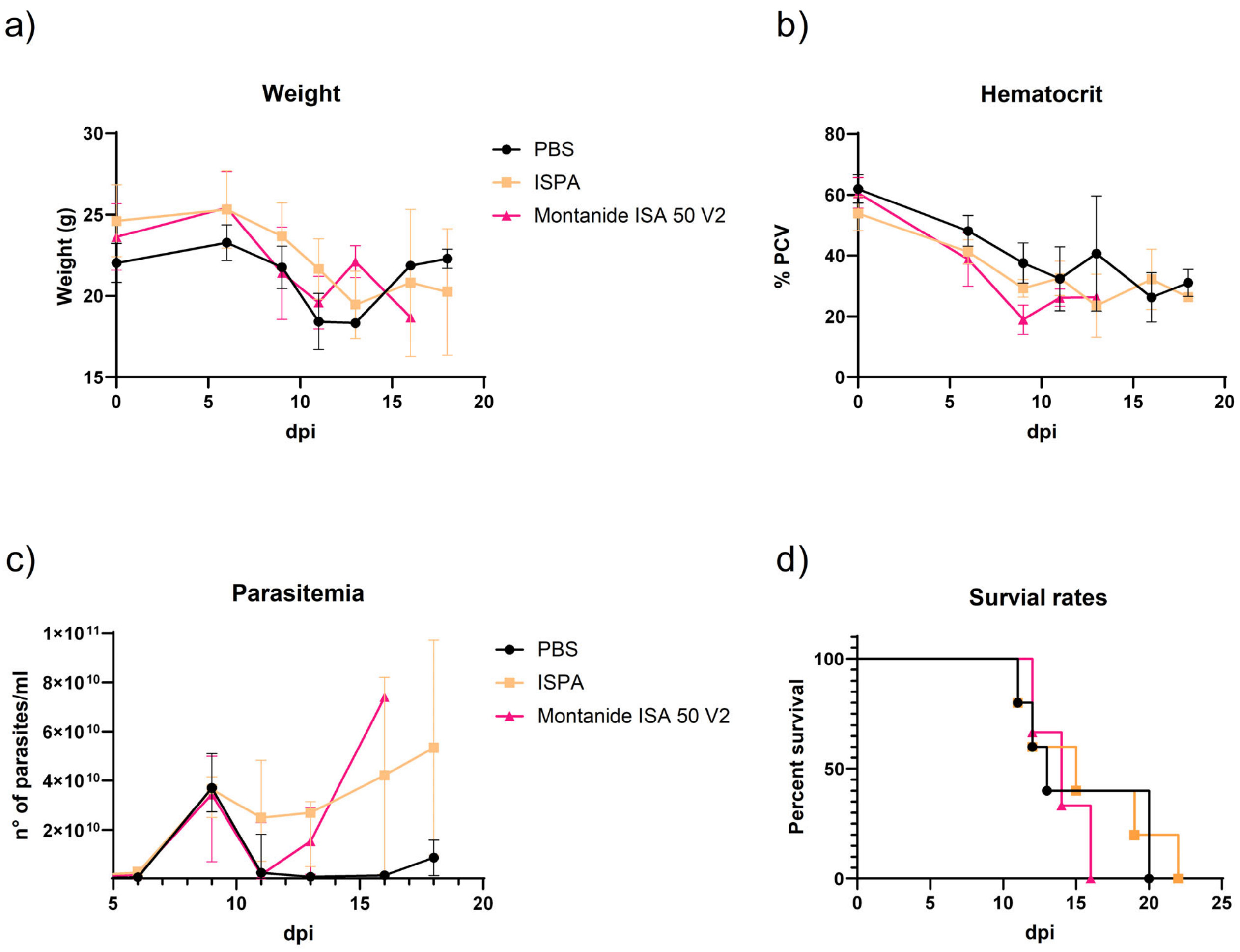

Figure S2. Effects of vaccination on parasitemia, anemia, body weight, and survival following experimental *T. vivax* infection in controls groups. BALB/c mice immunized with ISPA, Montanide, or PBS (n = 5/group) were challenged intraperitoneally with 1000 bloodstream forms of the *T. vivax* Y486 strain 14 days after the final immunization. (a) body weight, (b) hematocrit expressed as packed cell volume (PCV), (c) parasitemia, and (d) survival rates. Data shown correspond to one representative experiment of three performed independently.
